# Reverse takotsubo cardiomyopathy: two case reports and review of the literature

**DOI:** 10.1186/1752-1947-7-84

**Published:** 2013-03-19

**Authors:** Gautam R Patankar, James W Choi, Jeffrey M Schussler

**Affiliations:** 1 Department of Internal Medicine Suite H-102, c/o Suzanne Watts - Program Coordinator, Baylor University Medical Center, 3500 Gaston Avenue, Dallas, TX 75246, USA; 2 Baylor University Medical Center, 621 Hall Street #400, Dallas, TX 75226, USA; 3 Baylor University Medical Center, 621 North Hall Street Suite 500, Dallas, Texas 75226, USA; 4 Texas A&M Health Science Center, College of Medicine, Dallas, Texas, USA

## Abstract

**Introduction:**

Reverse takotsubo cardiomyopathy is a rare variant of classic takotsubo cardiomyopathy that presents within a different patient profile and with its own hemodynamic considerations. Its recognition is important for prognostic, evaluation and treatment considerations.

**Case presentation:**

Case 1: A 69-year-old Caucasian woman presented with substernal chest pain following a motor vehicle accident. During her evaluation, she was found to have positive results for cardiac enzymes and underwent left heart cardiac catheterization. The results of the catheterization demonstrated no significant coronary stenosis. However, her ventriculogram showed basal and anterior akinesis.

Case 2: A 62-year-old Caucasian woman began having substernal chest pain that radiated to her shoulder blades. She was taken to a local area hospital where she was found to have elevated troponins. A left heart catheterization showed an ejection fraction of 35% with hypokinesis of the anterior and posterobasal walls of her heart, with 30% stenosis of her left anterior descending artery but no other significant coronary artery stenosis.

**Conclusion:**

The cases in this report illustrate a lesser-known variant of takotsubo cardiomyopathy.

## Introduction

Takotsubo cardiomyopathy is typified by a transient systolic dysfunction of the apical segments of the left ventricle mimicking myocardial infarction in the absence of obstructive coronary artery disease. The pattern is also described as apical ballooning. It accounts for approximately 1.2% of all troponin proven acute coronary syndrome events [1]. Reverse takotsubo, a variant form of takotsubo cardiomyopathy in which the basal and midventricular segments of the left ventricle are akinetic, occurs in a minority of patients [1]. The majority of takotsubo cardiomyopathy patients recover cardiac function within three to six months. We present the case of two patients who had reversible takotsubo cardiomyopathy and offer a review of the literature regarding mechanism of injury, risk profile and clinic presentation.

## Case presentation

### Case 1

A 69-year-old Caucasian woman with a past medical history of atrial fibrillation presented to Baylor University Medical Center, Waxahachie, TX, USA after having substernal chest pain following a motor vehicle accident. Her initial electrocardiogram showed atrial fibrillation with a heart rate of 60 to 70 beats per minute with no S-T-wave changes. Her initial level of troponin was 0.85ng/mL and trended up to a peak level of 4.58ng/mL. She was loaded with clopidogrel bisulfate (Plavix®) started on a heparin drip and taken to emergent cardiac catheterization at Baylor University Medical Center, Dallas, TX, USA.

Catheterization showed her to have a depressed left ventricular ejection fraction of 40% with moderate inferior and anterolateral hypokinesis with a 30% mid stenosis of her right coronary artery but otherwise unremarkable coronaries. She was discharged home on metoprolol tartrate (Lopressor®) aspirin and ramipril. Our patient’s subsequent transthoracic echocardiogram demonstrated normal left ventricular systolic function with an estimated ejection fraction of 55%.

### Case 2

The second patient, a 62-year-old Caucasian woman with a history of atrial fibrillation and atrial flutter (status post transseptal ablation in 2005) began having substernal chest pain that radiated to her shoulder blades. No emotional stressors preceded this pain. She was taken to a local area hospital where she was found to have new T-wave inversion in leads V1 and V2 and an initial elevated level of troponins of 6.22ng/mL that later increased to 8.85ng/mL.

**Figure 1:**
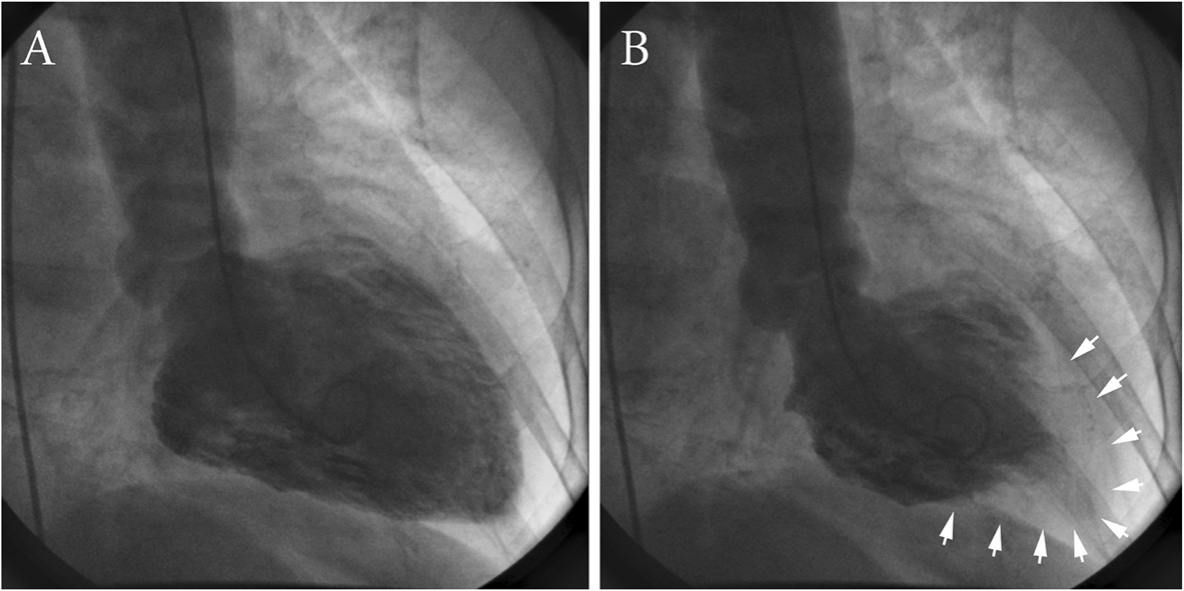
**Left ventriculogram of a patient with reverse-takotsubo cardiomyopathy in diastole (panel A) and systole (panel B).** Arrows denote the areas of normal ventricular movement in the apical segments. The basal segments remain hypokinetic. This is in contrast to typical takotsubo syndrome, where there is “apical ballooning,” and the apical segments remain hypokinetic.

A left heart catheterization showed an ejection fraction of 35% with hypokinesis of the anterior and posterobasal segments of her heart (Figure 1, Additional file 1), and a 30% stenosis of her left anterior descending artery, but no other significant coronary artery stenosis. She was discharged on lisinopril (Zestril®), simvastatin (Zocor®) and aspirin. A repeat echocardiogram one month later showed an ejection fraction of 55% to 60% and no wall motional abnormalities.

## Discussion

Takotsubo cardiomyopathy, also known as broken heart syndrome, is a cardiomyopathy typically seen in the setting of acute medical illness or emotional or physical stress, and is thought to be caused by a catecholamine-mediated injury [2]. The pathologic role of catecholamines has been suggestive, as there have been cases in which endogenous adrenergic stimulation (for example, iatrogenic or via a pheochromocytoma) has resulted in the manifestation of this entity [3]. The exact mechanism of injury caused by catecholamines is less well understood. There are two primary theories - vascular dysfunction and catecholamine-induced toxicity.

Vasospasm is strongly associated with takotsubo cardiomyopathy. Patients undergoing angiography have demonstrated multifocal coronary vasospasm that results in a pattern of apical ballooning [4]. Patients have also had abnormal thrombolysis in myocardial infarction frame counts on angiography in the absence of obstructive coronary disease, suggesting impairment of epicardial coronary circulation due to microcirculatory spasm [5].

Endomyocardial biopsy data in patients with takotsubo cardiomyopathy suggest that myocyte injury occurs in the presence of excessive catecholamines [6]. Histologic findings from patients with takotsubo cardiomyopathy document myofibrillar degeneration, contraction band necrosis and mononuclear leukocyte infiltration, which are forms of myocyte injury witnessed in catecholamine toxicity [7]. Molecular studies have shown that high doses of epinephrine are directly toxic to the cells, causing a rise in adenosine 3':5'-cyclic monophosphate and calcium levels that then trigger the formation of free oxygen radicals, the initiation of expression of stress response genes, and induction of apoptosis [8].

Compared with patients who have apical ballooning, patients with reverse takotsubo cardiomyopathy present at a younger age, with a mean age of 36, and often they have an emotional or physical stress trigger [9,10].

The belief is that catecholamines act on adrenoreceptors that have their highest density within the apex of the heart in postmenopausal women, which explains the occurrence of the apical variant in older women [8]. The presentation of inverted takotsubo cardiomyopathy in younger patients may be due to the abundance of adrenoreceptors at the base of the heart, compared with in the apex in older patients.

Patients with reverse takotsubo cardiomyopathy may present with less pulmonary edema, dyspnea and cardiogenic shock than patients with classic takotsubo cardiomyopathy [9]. This finding suggests that the differences in the clinical features are concomitant with possible hemodynamic changes caused by differences in the location of regional wall motion abnormality.

In classic takotsubo cardiomyopathy, left ventricular outflow tract obstruction from left ventricular basal hyperkinesis can contribute to shock or mitral regurgitation, or pseudo-hypertrophic cardiomyopathy can explain the hemodynamic profile [11].

Patients with reverse takotsubo cardiomyopathy have been reported to have significantly higher levels of cardiac markers, such as creatine kinase M (muscle type) or B (brain type) and troponin-I, than patients with apical or midventricular takotsubo cardiomyopathy [10]. This could be explained by the extent of myocardium involved in each form, with more myocardial tissue being affected in reverse rather than classic takotsubo cardiomyopathy.

## Conclusion

Reverse takotsubo cardiomyopathy is a variant with similar pathophysiological causes as classic takotsubo cardiomyopathy but with a different patient profile and presenting symptoms. This rarer variant of takotsubo cardiomyopathy is important to identify as it tends not to be recognized as readily as the traditional presentation. Because it tends to have the same clinical course, its recognition can aid in the expectation of recovery and prognosis, which can help with regards to treatment plans.

## Consent

Written informed consent was obtained from the patients for publication of this case report and accompanying images. A copy of the written consent is available for review by the Editor-in-Chief of this journal.

## Competing interests

The authors declare that they have no competing interests.

## Authors’ contributions

JS and JC identified the patients and performed the catheterization on each of the patients. GP and JS were major contributors in writing the manuscript. All authors read and approved the final manuscript.

## Supplementary Material

Additional file 1 **Cine ventriculography demonstrating a pattern of ventricular systole consistent with reverse-takotsubo cardiomyopathy.** The basal segments are hypokinetic, while the apex moves in systole. Click here for file
